# Feasibility of High‐Density Surface Electromyography for the Detection of Neuromuscular Disorders in Children

**DOI:** 10.1002/mus.70168

**Published:** 2026-02-03

**Authors:** Eduardo Martinez‐Valdes, Ignacio Contreras‐Hernandez, Ragul Selvamoorthy, Francesco Negro, Andrew Lawley

**Affiliations:** ^1^ School of Sport, Exercise and Rehabilitation Sciences, College of Life and Environmental Sciences University of Birmingham Birmingham UK; ^2^ Department of Clinical and Experimental Sciences Universita degli Studi di Brescia Brescia Italy; ^3^ Department of Neurophysiology Birmingham Children's Hospital Birmingham UK

**Keywords:** motor neuron, motor unit, myopathy, neuropathy, pediatric

## Abstract

**Introduction/Aims:**

Diagnosing neuromuscular disorders in children is challenging. Concentric needle electromyography (CNEMG) is the standard for electrophysiological assessments but has limitations in pediatric populations. High‐density surface electromyography (HDsEMG) provides a noninvasive technique with superior spatial resolution, enabling the identification and analysis of motor unit (MU) firing dynamics throughout the entire period of MU activity. This study assessed the feasibility of HDsEMG MU decomposition in children and explored parameters that differentiate neuropathy, myopathy, and normal findings.

**Methods:**

One hundred children (mean age 9.1 years, standard deviation [SD] 5.1) underwent CNEMG followed by HDsEMG. EMG signals were decomposed into individual MU spike trains, and MU yield, as well as firing properties (mean discharge rate [MDR], discharge rate variability [DRV]) were analyzed across diagnostic groups. Furthermore, correlations were assessed between MU action potential parameters obtained from CNEMG (MU amplitude and duration) and those obtained from HDsEMG.

**Results:**

MUs were reliably identified in 86.0% of children, with an average of 7 (4.2) MUs per participant. Among MU firing parameters, DRV was significantly higher in children with myopathy (*p* = 0.005). Additionally, MU duration from HDsEMG correlated weakly with CNEMG values (*r* = 0.31) and successfully discriminated myopathy from normal and neuropathic groups (*p* = 0.02).

**Discussion:**

HDsEMG MU decomposition is feasible in children with neuromuscular disorders, providing valuable insights into MU firing and MU action potential properties. This technique has the potential to improve diagnosis and monitoring of pediatric neuromuscular conditions. Nevertheless, further signal processing refinements are warranted to enhance its discriminative capacity for detecting neuromuscular disorders in children.

## 1 Introduction

Electrodiagnostic studies can be a useful diagnostic tool for the evaluation of neuromuscular disorders in children [1], although with changes in referral patterns reported in association with major advances in genetic analysis [2].

Identifying myopathy in young children can be challenging, as the reported diagnostic sensitivity of needle electromyography (EMG) varies widely, ranging from 10% to 86% [3, 4, 5, 6]. This may reflect variations in study populations, EMG analysis techniques, and operator experience. Quantitative motor unit action potential (MUAP) analysis may improve diagnostic sensitivity [3], although limitations still exist. First, needle EMG examination may be uncomfortable for young children [7]. Second, and related to the first point, EMG examination in young children typically involves fewer needle insertions than in adults, which may miss patchy distribution of muscle involvement. Third, normal MUAPs in infants have shorter durations compared to adults, reflecting smaller diameter of muscle fibers and endplate zones [8]. This can make it challenging to distinguish between pathological and normal MUAPs in infancy, even with quantitative MUAP analysis techniques.

Techniques to improve electrodiagnostic studies in young children would ideally be noninvasive, allow increased sampling of both the number of muscles that can be studied and the area of individual muscles, and potentially allow analysis of parameters other than those related to MUAP morphology. High‐density surface electromyography (HDsEMG) is widely used in physiological sciences, sports, and neuroscience research. Previous investigations from a single center reported inconclusive results with this technique in children with neuromuscular disorders [9, 10, 11]. However, there have been considerable recent advances in HDsEMG, particularly motor unit identification with automatic EMG signal decomposition algorithms [12, 13, 14] and the reliability and reproducibility of the technique has been demonstrated in adults [15].

The aims of this study are, firstly, to explore the feasibility of recording HDsEMG in children in a clinical setting, and secondly, to explore the potential diagnostic utility of HDsEMG by comparing motor units decomposed from HDsEMG recordings to those recorded with needle EMG.

## 2 Methods

### 2.1 Participants and Procedures

This was a prospective study performed over a 12‐month period between May 2023 and May 2024 in the Clinical Neurophysiology department at Birmingham Children's Hospital. All children (age 0–16 years) referred for electrodiagnostic studies with a clinical question of an underlying neuromuscular disorder were eligible for inclusion. Exclusion criteria included children with skin lesions or a history of allergy to materials used for recording HDsEMG. Informed written consent was obtained to perform HDsEMG recordings in addition to standard electrodiagnostic studies. To explore patient experience, all children who could understand necessary instructions were asked to complete a revised Faces Pain Scale [16] for both needle EMG and HDsEMG, with a score of 0 indicating no pain and 10 indicating maximum pain. Ethical approvals were granted from the University of Birmingham research governance department and from the NHS Health Research Authority (IRAS ID: 319384).

### 2.2 Electrophysiology—Concentric Needle EMG (CNEMG)

Nerve conduction studies and CNEMG examination were performed by the same investigator (A.L.). No sedation was used. Temperature was checked and warmed to at least 30°C in lower limbs and 32°C in upper limbs. Muscle selection for CNEMG examination was guided by presenting symptoms, patient age, and tolerance of the test, with tibialis anterior and biceps brachii most frequently examined. EMG was performed using a 30G Ambu Neuroline Concentric needle electrode and recorded with Sierra Summit software version 4 (Cadwell Inc., Kennewick, WA, US), with filter settings of 5 Hz to 10 kHz. Multi‐MUAP analysis was performed offline using automated software. At least 5 individual MUAPs were required to confirm a MUAP was unique, and MUAPs with amplitude less than 100 μV were excluded from analysis. MUAP analysis was performed at 200 μV/Div, and automated placement of cursors was accepted unless clearly incorrect. Overall diagnostic conclusion was based on a combination of qualitative and quantitative assessment, particularly measurement of MUAP duration with multi‐MUAP analysis. CNEMG findings were classified as normal, myopathic, or neuropathic.

### 2.3 Electrophysiology—HDsEMG

HDsEMG was recorded using a 64‐electrode grid, with 4 or 8 mm interelectrode distance (IED) depending on muscle size, placed over the muscle of interest. AL received HDsEMG training from E.M.‐V. A.L. performed all measurements in the clinic. HDsEMG was sampled at 2000 Hz and converted to digital data by a 16‐bit analogue to digital converter (64‐channel EMG amplifier Sessantaquattro, OT Bioelettronica, Torino, Italy). Electrodes were attached using conductive paste to create good contact between skin and electrodes. Recordings were made using OT Biolab+ software and later analyzed with MATLAB (The Mathworks Inc., Natick, Massachusetts, USA). HDsEMG was initially recorded during spontaneous movements, in the same way that needle EMG recordings were obtained. Subsequently, where children were old enough to follow simple instructions, a videogame EMG biofeedback app (OT Bioelettronica Planes, Torino, Italy) was used to standardize level of muscle contraction against manual resistance. The app uses the amplitude of the envelope of the interference pattern during maximal volitional contraction of the muscle. Therefore, the child was first asked to perform three maximal voluntary contractions, and the peak EMG value (50 ms window) was used as a reference for submaximal contractions. Feedback EMG was recorded in a bipolar configuration (16 mm IED) using the central channels of the electrode grid. After the maximal contraction assessment, the child was instructed to use a virtual plane to collect virtual coins displayed on the screen. The task included a 2‐s ramp‐up phase, a 20‐s hold at 20% of the maximal EMG, and a 2‐s ramp‐down phase. This procedure allowed stable HDsEMG signals, without the need for additional dynamometers or force transducers.

### 2.4 HDsEMG Signal Analysis

The HD‐sEMG signals recorded during the isometric contractions were decomposed into motor unit spike trains with an algorithm based on blind source separation, which provides automatic identification of multiple single motor units [14]. Single motor units were assessed for decomposition accuracy with a validated metric (Silhouette, SIL), which was set to > 0.80. SIL is a normalized measure of the relative height of the peaks of the decomposed spike trains with respect to the baseline noise and it is related to the sensitivity of the decomposition [17]. In this study, the SIL value was set at a lower threshold compared to previous studies in healthy adults since data in pathological individuals may exhibit unphysiological firing patterns that could be potentially discarded by the decomposition algorithm if a high SIL threshold is used [17].

Unlike CNEMG, which analyzes MUAP morphology based on the average MUAP from a minimum of five firings, HDsEMG allows for the decomposition of signals throughout the entire contraction, enabling assessment of motor unit firing patterns for the full duration of unit activity. Discharge times of the identified motor units were converted into binary spike trains, and firing properties were analyzed using instantaneous firing rate profiles (calculated as the inverse of the interspike interval: 1/interspike interval). The mean discharge rate and discharge rate variability (quantified as the coefficient of variation for discharge rate [CVDR], defined as SD discharge rate/mean discharge rate × 100) were assessed. These values were computed at the time point when participants were able to maintain a stable EMG level during the contraction. In addition, parameters such as recruitment and de‐recruitment thresholds were calculated. Recruitment and de‐recruitment thresholds were defined as the % of maximum EMG level where motor units began and ceased firing action potentials during the ramp‐up and ramp‐down phases of the contraction respectively.

Since HDsEMG decomposition algorithms can introduce errors in firing pattern identification—such as missing pulses leading to abnormally long interspike intervals [ISI]—all motor unit discharges were visually inspected and corrected when necessary. Additionally, merged motor units (where the algorithm erroneously classified two distinct units as one, combining their firing activity) were identified and excluded from analysis when detected [17]. However, the quality control process was less conservative than in studies on healthy individuals, as pathological firing patterns may naturally include irregular spiking intervals or prolonged pauses in action potential firing [18]. All the editing procedures were conducted according to the latest consensus statement in single motor unit recordings [17].

Besides the analysis of motor unit firing data, HDsEMG MUAP morphology parameters such as MUAP amplitude and duration were assessed and compared between HDsEMG and CNEMG, given that these values are commonly used in clinical practice. HDsEMG MUAP amplitude values were assessed by calculating the average root mean square and peak to peak values of all EMG channels of the electrode grid in bipolar‐single differential derivation (59 channels). The duration of HDsEMG MUAPs (half‐duration) was estimated by measuring the time interval between the onset of the MUAP rise (depolarization) and its peak, or between the negative peak and the end of the MUAP (repolarization) when the MUAP polarity was inverted. This value was also averaged across the 59 bipolar channels of the electrode grid.

All motor unit data was recorded, analyzed, and reported according to the consensus for experimental design in EMG: single motor unit matrix [17].

### 2.5 Statistical Analysis

All continuous variables are presented as mean (standard deviation, SD). Demographic variables (e.g., age, weight), CNEMG motor unit characteristics (number of identified units, mean duration [ms], mean amplitude [μV], and polyphasic percentage [%]), and HDsEMG motor unit parameters (mean discharge rate, CVDR, MUAP amplitude, and duration) were compared across groups (normal, myopathy, and neuropathy) using one‐way analysis of variance (ANOVA). If ANOVA results were significant, pairwise post hoc analyses were made with a Tukey test.

Differences in discomfort levels between CNEMG and HDsEMG (assessed using the Revised Faces scale) were analyzed using a paired *t*‐test. Associations between CNEMG and HDsEMG MUAP parameters (amplitude and duration) were examined using the Pearson correlation coefficient (*r*) and linear regression. Correlations are categorized as weak (0.10–0.39), moderate (0.40–0.69), or strong (0.70–0.89) according to [19].

Motor unit firing parameters were compared across groups using data from the entire study population (*N* = 96, see participants section below). However, comparisons of MUAP amplitude and duration were restricted to children who used an 8 mm IED grid (*N* = 78), as variations in IED spacing can influence these parameters.

Statistical significance was set at an *α* level of 0.05, and all analyses were performed using Prism version 10.0 (GraphPad Software, San Diego, CA, USA).

## 3 Results

### 3.1 Participants

110 potential participants were identified, with 100 consenting for HDsEMG recordings. Participant age ranged from 11 days corrected age to 16 years, with tibialis anterior most commonly examined (Other examined muscles can be seen in Table 1). Diagnostic findings of the neurophysiology study were normal for 62 participants, with 19 having myopathic EMG abnormalities, 15 having neuropathic EMG abnormalities, and 4 showing mixed or inconclusive findings. These four participants were not included in the analysis. Full participant demographics and CNEMG results for 96 participants are presented in Table 1.

**TABLE 1:** Participant characteristics and CNEMG results.

	Normal	Myopathy	Neuropathy	*p*
Number of participants	62	19	15	—
Sex	40 M/22 F	13 M/6 F	7 M/8 F	—
Age (y)	9.6 (5.2)	7.3 (4.2)	9.0 (5.4)	0.23
Height (cm)	138.2 (36.4)	124.3 (25.5)	132.4 (29.6)	0.32
Weight (kg)	41.9 (25.8)	30.5 (15.8)	35.7 (21.1)	0.18
Temperature (°C)	31.4 (1.3)	31.8 (1.4)	31.8 (1.1)	0.32
Examined muscle	BB = 11, TA = 51	BB = 3, TA = 16	BB = 1, TA = 11, AP = 1, ED = 2	—
**Motor units (*N*)**	**14.0 (6.3)**	**11.3 (5.7)**	**7.9 (4.4)**	**0.02**
**Mean duration (ms)**	**8.6 (1.0)**	**6.6 (1.5)**	**13.4 (4.1)**	**< 0.001**
**Mean amplitude, (μV)**	**512.8 (159.0)**	**409.9 (186.5)**	**1179.6 (750.7)**	**< 0.001**
**Polyphasic (%)**	**4.2 (5.3)**	**19.2 (22.4)**	**16.7 (22.6)**	**< 0.001**

*Note:* Results represent means (standard deviation, SD). Significant results are presented in bold.

Abbreviations: AP = adductor pollicis brevis, BB = biceps bracchi, ED = extensor digitorum, TA = tibialis anterior.

### 3.2 Participant Reported Discomfort

**FIGURE 1:**
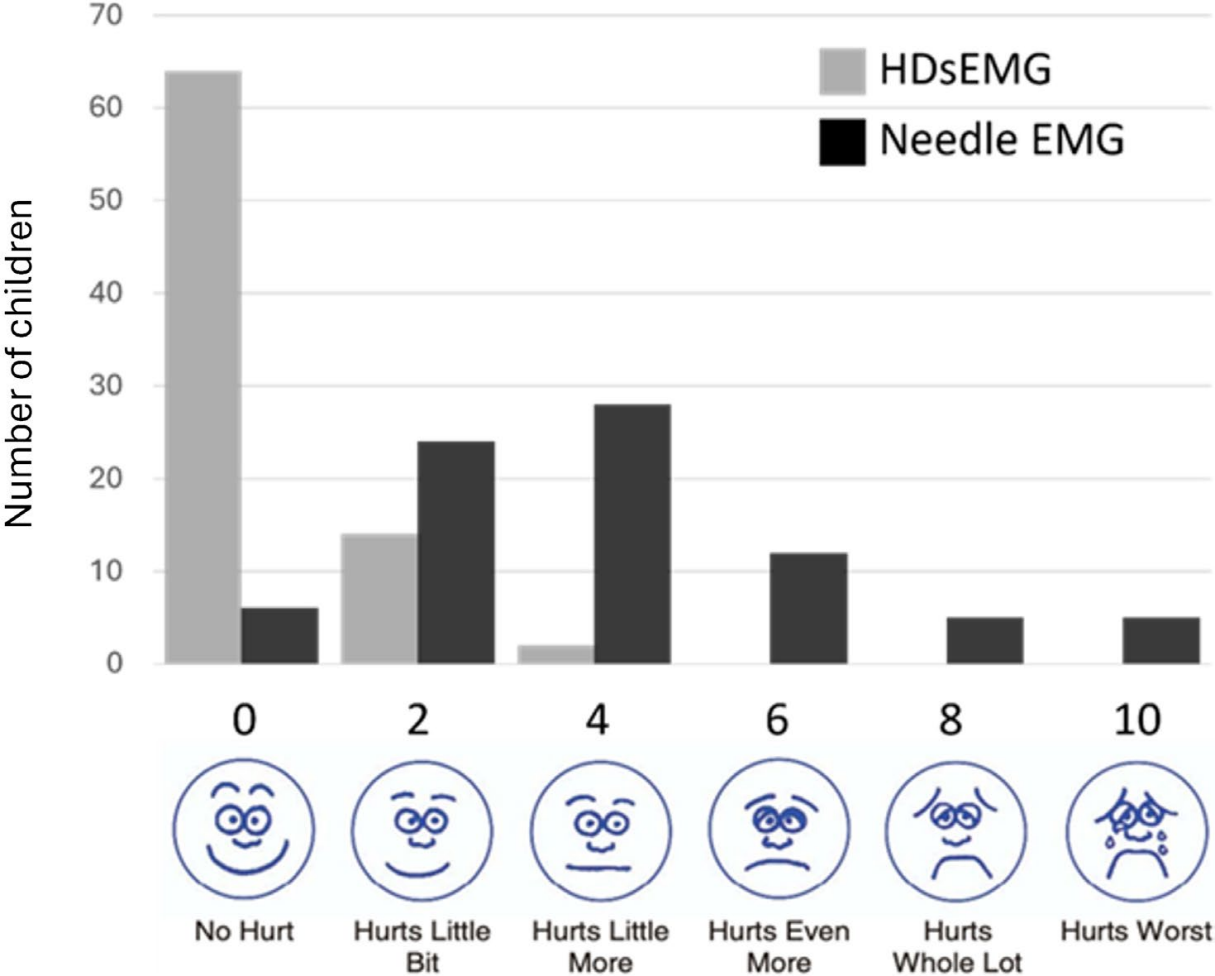
Revised faces pain scale response distribution for HDsEMG and CNEMG (*n* = 80).

Revised faces pain scales were completed by 80 participants (age 11.0 [3.8] years). Pain scores were significantly lower for HDsEMG compared with CNEMG examination (0.45 [0.95] vs 4.03 [2.5], *p* < 0.001). Only one participant reported a preference for CNEMG examination but reported a low level of discomfort for both procedures (4 vs. 2). Sixty four participants reported no discomfort with HDsEMG. Where discomfort was experienced, this was related to either electrode removal or continuous muscle contraction. Further details are provided in Figure 1.

### 3.3 HDsEMG Decomposition—Motor Unit Identification

**FIGURE 2:**
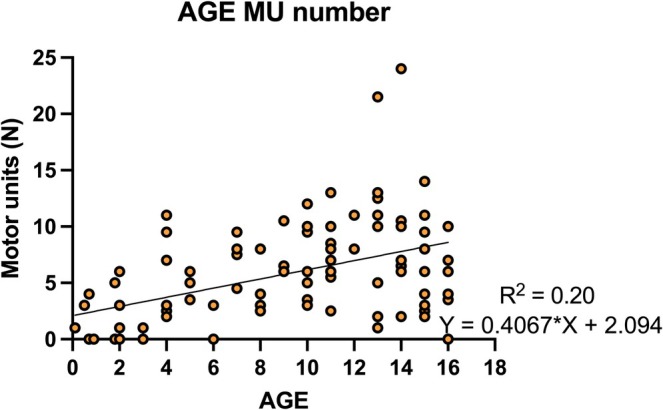
Association between participant age and number of motor units identified.

A total of 101 HDsEMG recordings were made in 100 participants. Eighteen recordings were made with 4 mm IED; the remainder with 8 mm IED. The mean value of force quantified via EMG feedback (20% of max EMG) and the variability of the EMG signal (CoV EMG) during the sustained contractions was not different between groups, confirming the maintenance of force level and control across groups (EMG amplitude: *p* = 0.503 and EMG variability: *p* = 0.216). Following HDsEMG decomposition, motor units with a full firing pattern (i.e., motor units active for more than 25% of total contraction time) could be identified on 86 children. From these children, 7 (4.2) motor units could be identified on average per participant. The ability of the decomposition algorithm to detect motor units varied between normal, myopathic and neuropathic groups, with the myopathy group being the group with the lower number of motor units identified (4.2 [5.4]) versus normal (7.4 [4.7]) and neuropathic (7.0 [3.2]), who had similar number of identified units (condition effect *p* = 0.034). Furthermore, the ability of the algorithm to detect motor units was moderately associated with the age of the participants (*r* = 0.447, *p* < 0.001) as the number of observed units increased with age (Figure 2).

### 3.4 HDsEMG Motor Unit Firing Analysis

**FIGURE 3:**
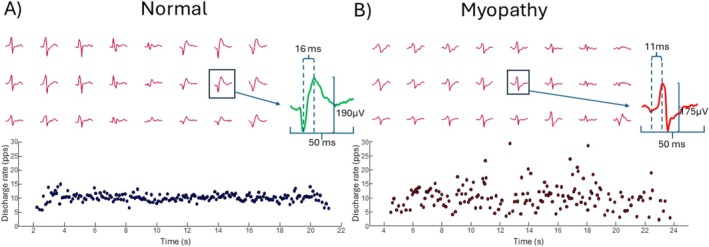
Representative MUAP spatial profiles and motor unit discharge patterns. (A) Individual with normal electrodiagnostic impression. MUAP profile is displayed above (24 channels). A single representative MUAP (green) with its half duration and peak to peak amplitude values can be seen on the right. Motor unit firing pattern can be seen below. When considering all 59 bipolar channels and the full pool of identified motor units, average MUAP half duration and peak to peak amplitude for this participant was 11.9 ms and 175.7 μV, respectively. (B) Individual with myopathy diagnostic impression. MUAP profile is displayed above (24 channels). A single representative MUAP (red) with its half duration and peak to peak amplitude values can be seen on the right. Motor unit firing pattern can be seen below. When considering all 59 bipolar channels and the full pool of identified motor units, average MUAP half duration and peak to peak amplitude for this participant was 14 ms and 190.2 μV, respectively. Despite the differences in MUAP amplitude and duration between these participants it is important to note the great variability in MUAP morphology across the electrode grid. Furthermore, note the more variable firing pattern for the individual with myopathy at the bottom right of the figure.

**FIGURE 4:**
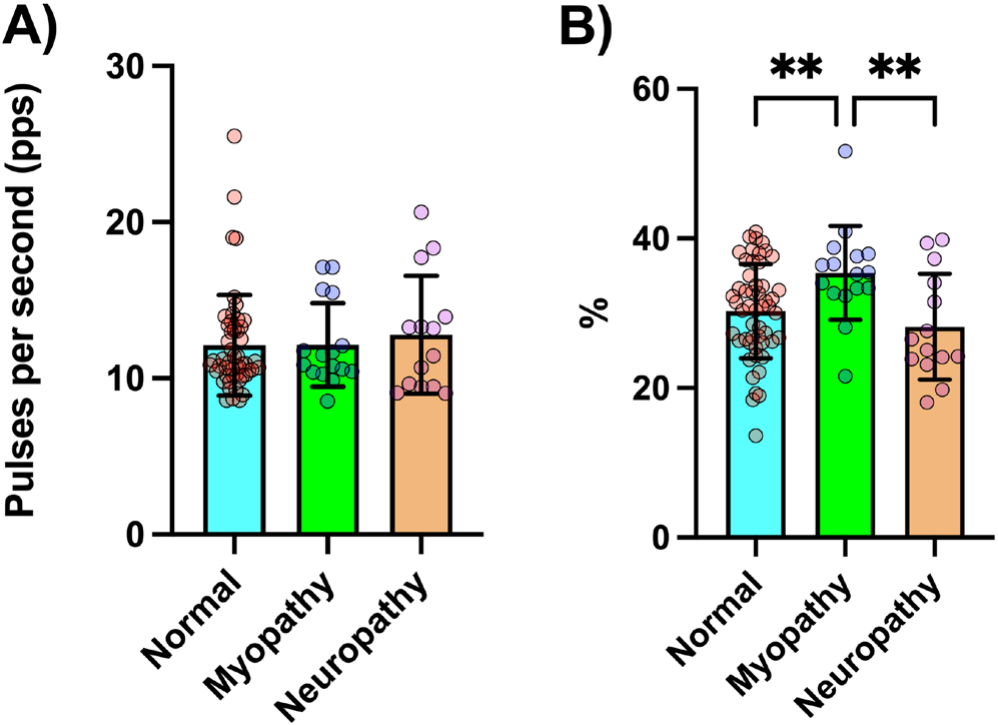
Motor unit firing results across the normal, myopathy and neuropathy groups. (A) Mean motor unit discharge rate. (B) Discharge rate variability quantified as the coefficient of variation in discharge rate. **p < 0.001.

Representative results of motor unit firing activity and MUAP morphology from a child with a normal electrophysiology test, as well as from a child with electrophysiological findings suggestive of myopathy, are shown in Figure 3. Motor unit recruitment and de‐recruitment thresholds were comparable across groups (*F* = 0.728, *p* = 0.486 and *F* = 0.750, *p* = 0.476, respectively), indicating that similar populations of motor units were assessed. Specifically, recruitment thresholds were 22.5% (16.7) MVC EMG for the normal group, 24.2% (15.5) MVC EMG for the myopathic group, and 21.6% (12.3) MVC EMG for the neuropathic group. Mean discharge rate showed no significant differences between groups (*F* = 0.402, *p* = 0.670). However, the CVDR was significantly higher in the myopathic group compared to both the normal and neuropathic groups (*F* = 5.7, *p* = 0.005). Motor unit firing results are presented in Figure 4.

### 3.5 Association Between HDsEMG and CNEMG Parameters

**FIGURE 5:**
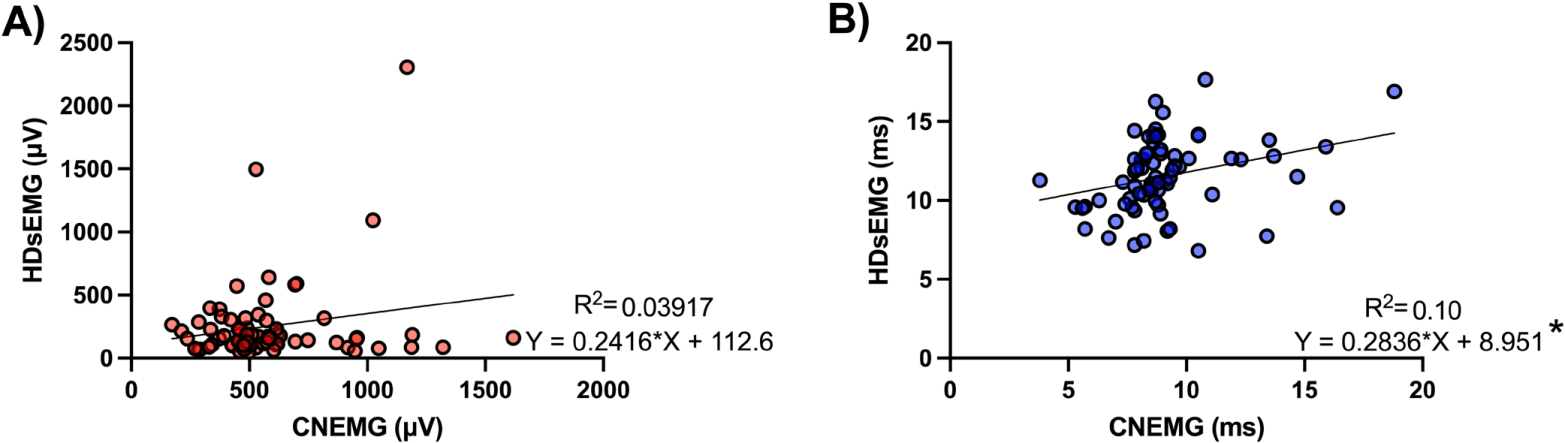
Association between MUAP parameters obtained from CNEMG and HDsEMG. (A) HDsEMG versus CNEMG MUAP amplitude. (B) HDsEMG versus CNEMG MUAP duration. Significant association (*p = 0.0086).

**FIGURE 6:**
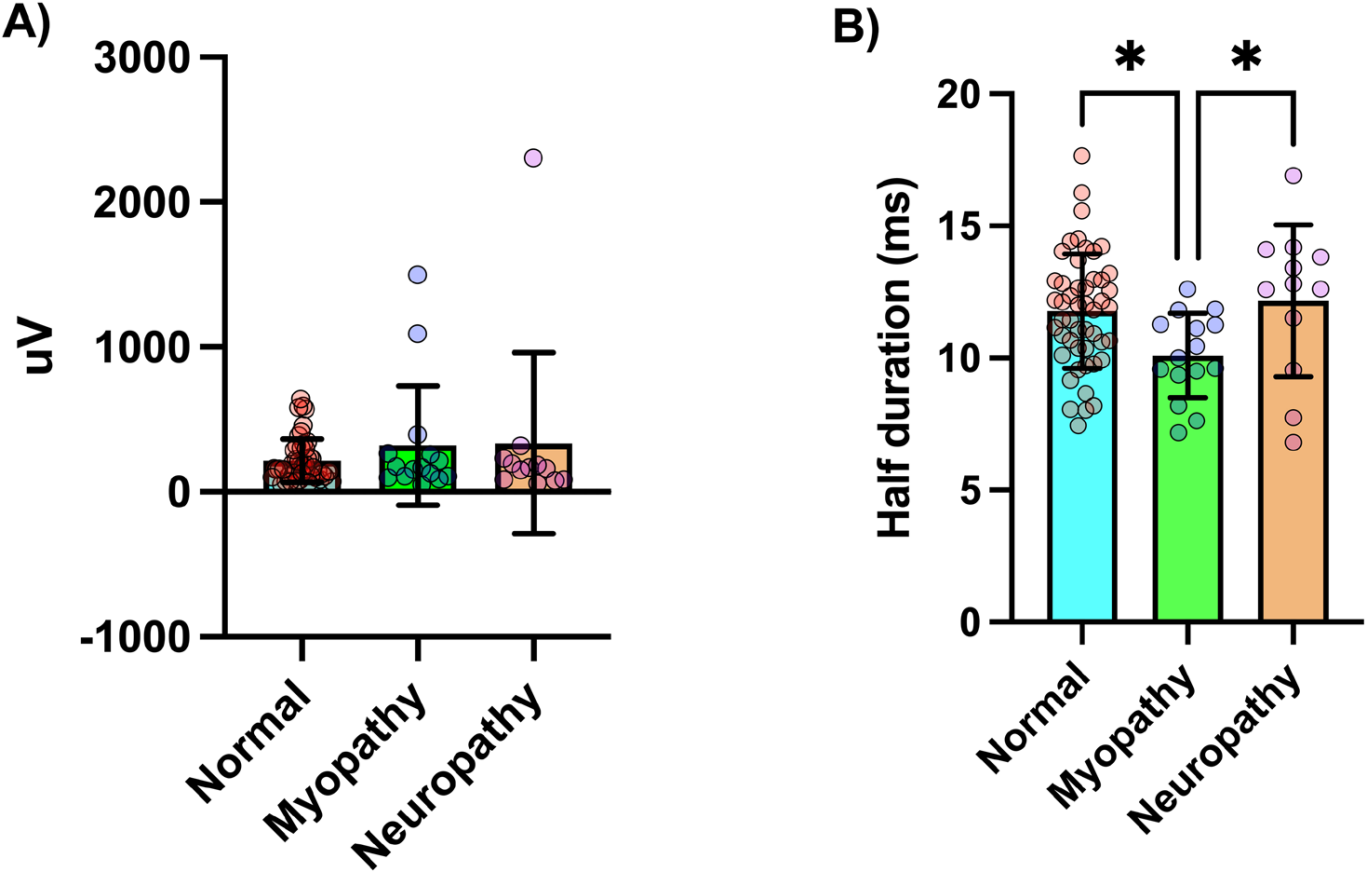
MUAP parameters from HDsEMG recordings compared across groups. (A) MUAP amplitude values (peak‐to‐peak MUAP values averaged across the whole electrode grid). (B) MUAP duration values (half‐length of the MUAP duration averaged across the whole electrode grid). Significant difference between groups, *p < 0.05.

MUAP amplitude values obtained from CNEMG recordings showed no significant correlation with HDsEMG MUAP peak‐to‐peak values (*r* = 0.20, *p* = 0.096). However, MUAP duration from CNEMG analysis was significantly but weakly correlated with MUAP half‐duration values from HDsEMG (*r* = 0.31, *p* = 0.0086). Figure 5 illustrates the association results between CNEMG and HDsEMG MUAP amplitude and duration. While HDsEMG MUAP amplitude values did not distinguish between groups (*F* = 1.038, *p* = 0.36), HDsEMG MUAP duration values were significantly shorter in the myopathic group compared to the normal and neuropathic groups (*F* = 4.024, *p* = 0.02). Comparisons of HDsEMG parameters across groups are presented in Figure 6.

## 4 Discussion

This study demonstrates that HDsEMG is feasible in children in a clinical setting. HDsEMG signals were successfully decomposed and enabled a detailed assessment of motor unit firing patterns. Children with myopathies exhibited greater firing variability compared with those showing normal or neuropathic electrophysiological profiles. Additionally, MUAP duration measured with HDsEMG effectively identified children with myopathies, although it showed only a weak correlation with CNEMG measurements. These results support HDsEMG as a promising noninvasive tool for motor unit analysis in pediatric populations.

### 4.1 Association Between CNEMG and HDsEMG

Our analysis revealed no association between MUAP amplitude measurements from CNEMG and HDsEMG. This is likely due to volume conduction effects, the low pass filtering effect of tissues, and the increased distance between recording electrodes and muscle fibers [20]. In contrast, CNEMG captures near‐fiber potentials more effectively, allowing for a more precise assessment of MUAP morphology [21]. We observed a significant correlation between MUAP half‐duration measured across the HDsEMG grid and MUAP duration obtained from CNEMG. Surface EMG action potentials inherently lose many characteristics of near‐fiber potentials, making it more difficult to detect polyphasic potentials, jitter, or jiggle effects. Nevertheless, certain MUAP morphological features—particularly duration—appear to be preserved in HDsEMG recordings. Due to challenges in accurately determining full MUAP duration across the entire electrode grid, we opted to quantify the time between the first positive turn and the MUAP peak or negative peak and end of the MUAP (end of repolarization) in the case of MUAPs with inverted polarity. Using this metric, we observed clear reductions in MUAP duration in the myopathy group, but we did not observe prolonged MUAP duration in the neuropathic CNEMG group, likely reflecting a small and heterogeneous sample. There was weak correlation between MUAP duration measured from HDsEMG and CNEMG‐derived values. This may be due to the substantial variability in MUAP morphology across the HDsEMG grid (see Figure 3). Future studies should aim to refine MUAP selection criteria by focusing on the most representative MUAPs for a given motor unit [22]. Additionally, more sophisticated analyses incorporating spectral content assessments and artificial intelligence techniques [23] could further enhance the ability to distinguish pathological changes in MUAP waveforms.

### 4.2 Benefits of HDsEMG Over CNEMG

Compared to CNEMG, HDsEMG is better tolerated and allows for the recording of larger muscle regions. Previous studies using HDsEMG in children have primarily relied on spatial analysis techniques to interpret interference EMG signal patterns [11]; these methods are limited by common challenges associated with sEMG signals [12]. Nevertheless, the high spatial sampling capability of HDsEMG, when combined with blind‐source separation techniques, enables the identification of individual motor unit firing potentials [14, 24], facilitating a more detailed investigation of motor unit firing dynamics during contractions.

Although quantitative MUAP analysis using CNEMG often reports larger sample sizes per contraction, this technique may average the MUAP template from small numbers of MUAPs. In addition, detailed assessment of motor unit firing properties in children is challenging due to difficulties in achieving a sustained contraction during CNEMG. In contrast, HDsEMG allows for both the assessment of motor unit firing properties and changes in MUAP morphology, thereby increasing the number of parameters that can be used to detect the presence of neuromuscular disorders.

### 4.3 Motor Unit Firing Activity and the Detection of Myopathies

The assessment of motor unit firing properties in neuromuscular disorders is rare, particularly in children. Among the few studies conducted in adults, findings have been inconsistent. For instance, increased discharge rates in myopathies and decreased rates in central disorders such as ALS have been reported [25], whereas others found no significant differences between conditions [26], consistent with the present study. One possible explanation for these discrepancies is the challenge of accurately tracking the same motor units during contractions using earlier methodologies. If a motor unit is not consistently identified, additional motor unit activity may be erroneously merged. This issue is particularly relevant in myopathies, in which motor units tend to be smaller and recruitment strategies may differ to compensate for muscle weakness [27]. Assessing motor unit recruitment remains challenging with any method, as motor unit identification depends largely on the volume of detectable motor unit activity within the electrode's recording field.

One of the most intriguing findings of this study was the increased variability in motor unit discharge rates in the myopathy group. Previous research using CNEMG has reported conflicting evidence regarding motor unit discharge variability in both neuropathic and myopathic conditions. For example, studies in adults have shown no change in firing variability in either condition [26] or increased variability only in neuropathic disorders [25]. These discrepancies may stem from methodological limitations, particularly in the ability to detect and track enough firings from a single motor unit. In contrast, our study allowed for the monitoring of a significant number of motor units and their firing behavior during contractions, revealing a clear increase in ISI variability in children in our myopathy group.

Potential mechanisms explaining this finding include impaired motor neuron excitability [28], reduced motor unit recruitment stability [27], and impaired neuromuscular transmission [29]. Changes in motor neuron excitability are unlikely to be the primary driver of increased firing variability since myopathies primarily affect muscle fiber properties. Instead, altered motor unit recruitment patterns may play a role, as individuals with myopathies often exhibit reduced motor unit innervation territories due to muscle fiber degeneration. This could lead to increased recruitment or spontaneous firing of the same motor unit to maintain force output. Additionally, acetylcholine receptor dysfunction at the neuromuscular junction can lead to failed or inconsistent transmission of action potentials, disrupting motor unit firing patterns [30].

### 4.4 Limitations

Some limitations should be considered. First, the analysis of HDsEMG was based on the interpretation of CNEMG electrophysiological findings rather than confirmed clinical diagnoses. Additionally, to enhance comparability, most recordings were conducted on the tibialis anterior muscle. Future studies should assess multiple muscles, particularly in conditions that affect muscle groups differently. Thirdly, in children capable of following instructions, surface EMG was used as feedback to regulate contraction force levels. Given the high variability of surface EMG in assessing maximal contraction intensity and force control, the use of a dynamometer would have provided more precise force measurements, ensuring consistency across participants. This would have also enabled comparisons of differences in strength between groups. Future studies should incorporate both HDsEMG and force measurements to better understand how motor unit function influences force generation in this population. Finally, the inclusion of ultrasound imaging could have helped to assess the influence of adipose tissue thickness and muscle architecture on motor unit variables [31].

### 4.5 Conclusions

HDsEMG motor unit decomposition is both feasible and well‐tolerated in children in a clinical setting, offering valuable insights into motor unit firing patterns and changes in MUAP morphology. Increased motor unit discharge rate variability and reduced MUAP duration were identified as potential indicators of myopathic disorders, although further work is required to confirm this finding. The findings of the present study do not indicate that HDsEMG can substitute for CN assessments. Nonetheless, the results provide a foundation for future methodological refinements aimed at enhancing its discriminative capacity for the detection of neuromuscular disorders in children.

## Author Contributions

**Eduardo Martinez‐Valdes:** conceptualization, investigation, funding acquisition, writing – original draft, methodology, validation, visualization, writing – review and editing, formal analysis, project administration, supervision, resources, data curation, software. **Ignacio Contreras‐Hernandez:** methodology, validation, visualization, writing – review and editing, data curation. **Ragul Selvamoorthy:** methodology, validation, visualization, writing – review and editing, data curation. **Francesco Negro:** methodology, writing – review and editing, formal analysis, software. **Andrew Lawley:** conceptualization, investigation, funding acquisition, writing – original draft, methodology, writing – review and editing, formal analysis, project administration, resources, data curation.

## Funding

This work was supported by Muscular Dystrophy UK, 22GRO‐PG12‐0572 and European Research Council, 101045605.

## Ethics Statement

We confirm that we have read the Journal's position on issues involved in ethical publication and affirm that this report is consistent with those guidelines.

## Conflicts of Interest

The authors declare no conflicts of interest.

## Data Availability

The data that support the findings of this study are available from the corresponding author upon reasonable request.
